# Advances in immunotherapy for mucosal melanoma: harnessing immune checkpoint inhibitors for improved treatment outcomes

**DOI:** 10.3389/fimmu.2024.1441410

**Published:** 2024-07-30

**Authors:** Zexing Shan, Fei Liu

**Affiliations:** ^1^ Department of Gastric Surgery, Liaoning Cancer Hospital and Institute, Cancer Hospital of China Medical University, Shenyang, China; ^2^ Department of Bone and Soft Tissue Tumor Surgery, Cancer Hospital of China Medical University, Liaoning Cancer Hospital & Institute, Shenyang, China

**Keywords:** mucosal melanoma, tumor microenvironment, immunotherapy, immune checkpoint inhibitors, cancer

## Abstract

Mucosal melanoma (MM) poses a significant clinical challenge due to its aggressive nature and limited treatment options. In recent years, immunotherapy has emerged as a promising strategy for MM, with a particular focus on immune checkpoint inhibitors such as PD-1 and CTLA-4 inhibitors. These inhibitors have demonstrated substantial efficacy by harnessing the body’s immune response against tumors. Moreover, adoptive cell transfer (ACT), anti-angiogenic therapy, and combination therapies have garnered attention for their potential in MM treatment. ACT involves modifying T cells to target melanoma cells, showing promising antitumor activity. Anti-angiogenic therapy aims to impede tumor growth by inhibiting angiogenesis, while combination therapies, including immune checkpoint inhibitors and targeted therapies, offer a multifaceted approach to overcome treatment resistance. This comprehensive review explores the advancements in immunotherapy for MM, highlighting the role of diverse therapeutic modalities in enhancing treatment outcomes and addressing the challenges posed by this aggressive malignancy.

## 1 Introduction

Melanoma, a malignancy arising from the transformation of melanocytes, which are cells originating from the neuroectoderm and responsible for melanin synthesis, presents as a malignant tumor characterized by early metastasis, increased invasiveness, and an unfavorable prognosis (1, 2). Over recent years, there has been a consistent rise in the annual incidence and mortality rates of melanoma. Mucosal melanoma (MM), originating from mucosal tissues, typically presents as solitary lentil-shaped cells adhering to the surface of mucosal squamous epithelial cells (3, 4). Despite its representation in only 1-5% of all melanoma cases, MM poses considerable challenges due to its heightened malignancy. Classification of MM is predominantly based on the primary site, which may include melanomas originating from the head and neck (nasopharynx and oral cavity), gastrointestinal tract (upper and lower digestive tract), urinary system, respiratory system, and other anatomical regions (5, 6). Mucosal melanoma (MM) is a rare subset of melanoma characterized by its lower incidence rate compared to cutaneous melanoma. Despite its infrequent occurrence, MM presents significant challenges due to its heightened malignancy and treatment complexities. However, there are reports suggesting a more favorable prognosis for MM patients. Zhang et al. conducted an analysis and found that MM patients had improved prognoses compared to Chinese cutaneous melanoma patients. This study made key observations regarding disparities in progression-free survival (PFS) between mucosal and cutaneous melanoma patients, the impact of age on MM prognosis, as well as the crucial role of staging and treatment regimens in cutaneous melanoma prognosis (7). MM can arise in various mucosal sites, including the respiratory, gastrointestinal, and genitourinary tracts. In contrast to cutaneous melanoma, MM demonstrates notable differences in epidemiology, etiology, and pathogenesis. The incidence rate of MM remains relatively stable and tends to occur at a later age compared to cutaneous melanoma, typically between 50 and 80 years (8). Additionally, MM has a higher incidence rate in females, particularly in vulvar and vaginal manifestations (9). The exact etiology and pathogenesis of MM are still unknown, but genetic factors are believed to play a critical role in its development. Unlike cutaneous melanoma, MM is rarely associated with BRAF gene mutations, while mutations and amplifications of the KIT oncogene are more common (3, 10). These genetic differences underline the unique makeup of MM and contribute to its heightened aggressiveness and poorer prognoses (11).

In recent years, the development of immunotherapy has provided optimism for patients contending with drug-resistant, recurrent metastatic tumors, and those lacking actionable driver genes (12, 13). Immunotherapy functions by stimulating and enhancing the inherent immune response of the body to combat the growth and spread of tumor cells (14, 15). Key immunotherapeutic strategies involve the deployment of immune cells, antibodies, and tumor vaccines to provoke an immune response in the body (16). In contrast to traditional treatment modalities, immunotherapy offers clear advantages. It has the ability to target the diversity of tumor cells, thereby overcoming challenges related to drug resistance and adverse effects (17). Moreover, the impact of immunotherapy can be enduring, establishing immune memory. Once the immune system has developed memory against tumor cells, it continuously monitors any remaining or recurring tumor cells (18). In recent times, the rise of immunotherapy using Immune Checkpoint Inhibitors (ICIs) has emerged as a promising approach to enhance the prognosis of individuals suffering from MM. This article aims to synthesize the current landscape and potential directions of immunotherapeutic strategies for MM, elucidating the mechanisms and roles of ICIs in the context of MM. Our primary goal is to offer new insights and pathways for the therapeutic care of patients with MM.

## 2 Pathogenesis and clinical characteristics of MM

### 2.1 The etiopathogenesis of MM

**Figure 1:**
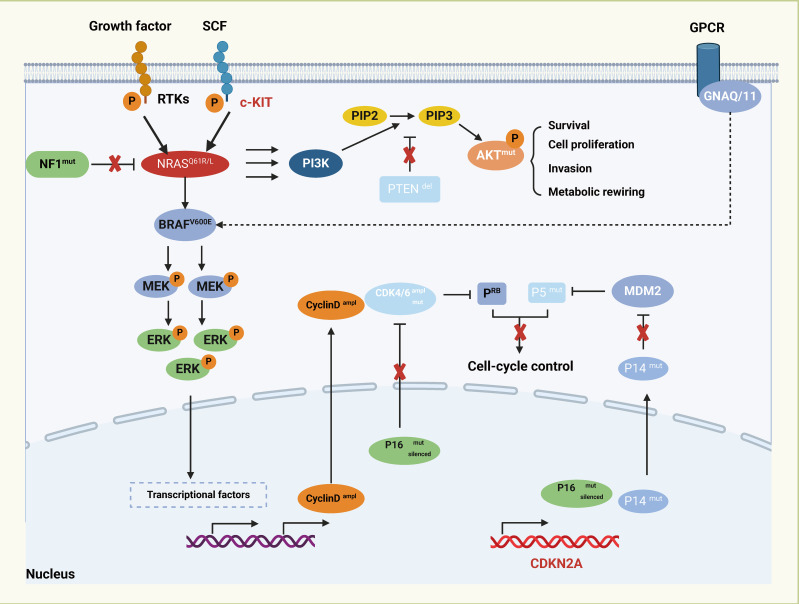
Major transcriptional regulators and signaling pathways in melanoma.

The pathogenesis of melanoma is a multifaceted process that involves the intricate interplay of various pathways, including genetics, environment, inflammation, immunity, and cellular signaling (**Figure 1**). Further exploration is necessary to fully elucidate the specific mechanisms underlying the disease. Although melanoma is primarily associated with skin exposed to ultraviolet radiation, it can also originate in different extracutaneous tissues where melanocytes are present, such as mucous membranes, ocular structures, and certain internal organs (19, 20). Racial disparities have been observed in the presentation of different subtypes of melanoma. Importantly, immunotherapy has demonstrated a favorable safety profile, harnessing the body’s innate defense mechanisms and resulting in relatively few severe adverse reactions (21). One notable breakthrough in the field of immunotherapy is the development of immune checkpoint inhibitors (ICIs). Functionally, ICIs counteract the immune suppression imposed by tumor cells, thereby restoring the immune competence of the body to combat the tumor (22). These therapies primarily target immune checkpoint proteins located on the surface of tumor cells, such as Programmed Death-1 (PD-1) and Cytotoxic T lymphocyte-associated protein 4 (CTLA-4). By binding to their respective ligands, ICIs intercept T cell activation signals, allowing tumor cells to evade immune surveillance (23, 24). Current investigations highlight the significant efficacy of ICIs in melanoma, non-small cell lung cancer, and bladder cancer (25–30). However, in other types of tumors, their effectiveness may be relatively limited or require combination with adjunct treatment modalities to achieve optimal outcomes. The unique physiological characteristics of these sites contribute to the inconspicuous onset and rapid deep-tissue and lymphatic spread of MM (31). Although mutations in KIT Proto-Oncogene, Receptor Tyrosine Kinase (KIT), Neuroblastoma RAS Viral Oncogene Homolog (NRAS), B-Raf Proto-Oncogene, Serine/Threonine Kinase (BRAF), neurofibromatosis type-1 (NF1), and Splicing Factor 3b, Subunit 1 (SF3B1) have been associated with MM development, their exact mechanisms remain incompletely understood. The current management strategies for MM primarily involve surgical intervention, chemotherapy, and radiotherapy (32). However, outcomes for advanced MM are dismal, with a less than 25% 5-year survival rate. Hence, there is an urgent need to elucidate the underlying pathogenesis and explore novel therapeutic approaches for MM.

The major transcriptional regulators and signaling pathways associated with melanoma pathogenesis, including the RAS-RAF-MEK-ERK cascade and the PI3K-AKT pathway, which are commonly activated in this cancer type either through mutations or other genetic alterations. The growth factors triggering receptor tyrosine kinases (RTKs) and the c-KIT receptor initiate the activation of the MAPK/ERK signaling cascade with NRAS and BRAF mutants. In the center, the diagram showcases the AKT signaling pathway connected to cell survival and metabolism, often dysregulated in melanoma through mutations such as PTEN deletion. G protein-coupled receptor (GPCR) signaling pathway has been implicated in melanoma through mutations in GNAQ or GNA11. Cell-cycle control is another major aspect of melanoma regulation, the cyclin-dependent kinase inhibitor CDKN2A encodes P16 and P14ARF proteins, both of which can be mutated, deleted, or silenced in melanoma.

### 2.2 MM in different systems

#### 2.2.1 Respiratory system

Respiratory mucosal melanoma (RTMM) is a rare neoplasm arising from melanocytes within the respiratory tract mucosa and can localize in various areas, including the nasal cavity, sinuses, oropharynx, larynx, trachea, and bronchi (33). Unlike cutaneous melanoma, RTMM displays distinctive etiological factors and clinical features (34). Genetic predisposition, chronic inflammatory responses, and prolonged viral infections have been suggested as potential etiological contributors to the development of RTMM (35). Furthermore, prolonged tobacco use and occupational exposures may increase an individual’s susceptibility to RTMM (35). Clinically, RTMM often presents insidiously, lacking early overt symptoms and demonstrating marked invasiveness. Its deep-seated location and inconspicuous clinical presentation commonly impede early detection, often leading to diagnosis at advanced stages. Patients may exhibit symptoms similar to other respiratory conditions, such as dyspnea, cough, hemoptysis, and hoarseness, thereby elevating the risk of misdiagnosis or delayed diagnosis (36). The diagnosis of RTMM primarily relies on histological examination and immunohistochemical profiling to ascertain tumor characteristics and origin. Given its invasive nature and proclivity for recurrence, conventional treatment approaches such as surgical resection, radiotherapy, and chemotherapy have shown limited efficacy (37). In recent years, immunotherapy has emerged as a promising therapeutic avenue. The development of immunotherapeutic agents, particularly immune checkpoint inhibitors (ICIs), has provided a degree of prognostic improvement for patients grappling with RTMM.

#### 2.2.2 Gastrointestinal system

Gastrointestinal mucosal melanoma is a rare and highly invasive malignancy that develops in the mucosal linings of the digestive tract, including the esophagus, stomach, small intestine, colon, and rectum (37, 38). Unlike cutaneous melanoma, the pathogenesis of gastrointestinal MM is believed to be complex, potentially involving genetic predispositions, chronic inflammation, viral infections, and dietary factors (39, 40). The disease often progresses discreetly, with minimal symptoms in its early stages, leading to advanced-stage diagnoses in many patients. Clinical presentations commonly include dyspepsia, abdominal discomfort, nausea, vomiting, and melena (41, 42), which can mimic symptoms of other gastrointestinal disorders, leading to misdiagnosis and delayed treatment. The diagnosis of gastrointestinal MM typically involves a tissue biopsy and immunohistochemical profiling to characterize the tumor and determine its origin, guiding treatment strategies and prognostic evaluations. Currently, conventional treatment modalities, such as surgery, radiotherapy, and chemotherapy, have limited effectiveness in managing gastrointestinal MM (43), highlighting the need for innovative approaches. In recent years, immunotherapy, particularly the use of immune checkpoint inhibitors (ICIs), has emerged as a promising therapeutic paradigm, significantly improving the antitumor efficacy of the host’s immune system and enhancing patient survival rates and prognoses.

#### 2.2.3 Urogenital system

Urogenital tract mucosal melanoma is evident within the mucosal tissues of various anatomical sites, including the urethra, bladder, cervix, vagina, testes, and prostate (6). In contrast to cutaneous melanoma, urogenital tract MM is closely associated with chronic inflammatory processes, genetic predispositions, exposure to carcinogens, and hormonal influences (44). Clinically, urogenital tract MM displays similarities with other melanoma subtypes, characterized by subtle progression, limited early symptoms, and marked invasiveness. Symptoms typically arise only in advanced stages of the disease, such as hematuria, urinary frequency, dysuria, and vaginal bleeding (45). These symptoms resemble those observed in various urogenital disorders, resulting in diagnostic challenges and delays in treatment. The diagnosis of urogenital tract MM primarily relies on histopathological evaluation and immunohistochemical profiling to determine tumor characteristics and origin (46, 47). Traditional treatment modalities, including surgical resection, radiotherapy, and chemotherapy, demonstrate limited effectiveness in managing urogenital tract MM (36). However, in recent years, immunotherapy has emerged as a promising therapeutic approach in this area. The introduction of immune checkpoint inhibitors (ICIs) and other immunomodulatory agents offers the potential to improve patient outcomes and survival rates to a certain extent.

### 2.3 The genetic landscape of MM

One of the prominent mutational drivers in melanoma is the BRAF gene, specifically the BRAF V600 mutation. This mutation is prevalent in melanoma, accounting for nearly half of all cases (48). The BRAF V600 mutation leads to sustained activation of BRAF kinase activity, resulting in abnormal stimulation of the Rapidly Accelerated Fibrosarcoma (RAF)/Mitogen-Activated Protein Kinase/Extracellular Signal-Regulated Kinase Kinase (MEK)/Extracellular Signal-Regulated Kinase (ERK) signaling cascade (49). Another significant mutational driver is NRAS, which is notably found in melanoma cases without BRAF mutations (48, 50). NRAS mutations activate proteins, initiating the Mitogen-Activated Protein Kinase (MAPK) signaling pathway and promoting cellular proliferation and survival (51). In mucosal malignant melanoma (MMM), Riobello et al. identified inactivation mutations and intragenic deletions of the NF1 gene, as well as activating mutations in the NRAS and KRAS genes. Cases with NF1 gene alterations showed reduced overall survival (OS) rates, indicating the potential prognostic and therapeutic relevance of these genetic alterations (52). Additionally, mutations in other genes such as Tumor Protein p53 (TP53), Cyclin-Dependent Kinase Inhibitor 2A (CDKN2A), KIT, and Phosphatase and Tensin Homolog (PTEN) are implicated in the etiology and progression of MM (3, 53–55). MM is characterized by reduced levels of T-cell infiltration and increased PD-L1 expression, indicating immune regulation dysfunction (56, 57). Genetic alterations in MM, including protein inactivation, mutagenesis, and proliferation-related mutations, play complex roles throughout the pathogenesis and evolution of the disease (58, 59). Given the intricacies of these genetic alterations, further comprehensive research is necessary to unravel their nuanced roles and therapeutic implications in MM.

### 2.4 Surgical and systemic treatment of MM

Due to its unique biological characteristics and clinical presentation, the management of Mucosal melanoma (MM) has consistently presented a significant challenge. Among the available treatment options, surgical resection is a widely used approach, particularly in cases of early-stage MM or individuals with a solitary resectable lesion (60). In localized MM cases, surgical intervention aims to remove the tumor along with the surrounding healthy tissue. This can be achieved through mucosal resection, mucosal resection with frozen section margin assessment, or margin clearance surgery, commonly performed in regions such as the oral and nasal cavities, genitalia, and digestive tract (61). For cases requiring more extensive excisions, surgical resection ensures the eradication of microscopic lesions around the tumor by removing the tumor along with a specified margin of adjacent healthy tissue (62). Heppt et al. conducted a study on MM in German patients, identifying the head and neck, female genital tract, and anorectal region as the primary sites, with anorectal MM having the worst prognosis (5). Additionally, NRAS, KIT, and BRAF gene mutations were found to be uniformly distributed. Male gender, advanced tumor stage, lymph node involvement, and incomplete resection were identified as risk factors for disease progression, with anorectal MM having the bleakest prognosis in European populations.

In cases where adjuvant radiotherapy is administered prior to surgical resection, surgery is usually performed to control local lesions or as a supplement to subsequent therapeutic interventions. Radiotherapy is commonly used as an adjuvant treatment in MM management, especially for postoperative lesion control or when surgery is not feasible. Although radiotherapy effectively targets cancer cells, it can cause damage to surrounding normal tissues. Chemotherapy is a systemic treatment option for advanced or metastatic MM, aimed at relieving symptoms and slowing tumor progression. In cases where surgery is not feasible, chemotherapy may be the primary therapeutic approach. Adjuvant chemotherapy can be administered before or after surgery to reduce tumor size, control lesions, decrease the risk of recurrence, or improve surgical success rates (63). Dacarbazine and vinblastine are commonly used chemotherapeutic agents in MM, acting by disrupting DNA synthesis or the division process of cancer cells. Combination chemotherapy, involving the simultaneous administration of multiple drugs like dacarbazine with vinblastine or oxaliplatin, is also utilized. Genetic profiling can identify individuals who may benefit from targeted therapy, which interferes with specific signaling pathways involved in cancer cell growth. Targeted therapy options include inhibitors of BRAF and MEK (64). Nevertheless, the high propensity of MM for recurrence and metastasis emphasizes the growing importance of systemic treatment approaches. Immunotherapy and targeted therapy have emerged as focal points of research efforts for individuals with locally advanced or metastatic MM. Immunotherapy uses drugs to enhance the patient’s immune response against MM (65). Flukes et al. conducted an analysis of patients with sinonasal MM (SNMM), highlighting surgical resection as the primary treatment modality, with a notable shift towards the adoption of endoscopic surgery over time. Radiotherapy was frequently utilized, and some patients showed benefit from immune checkpoint inhibitors (ICI) with partial or complete responses. The study emphasized the challenges and poor prognosis associated with SNMM, underscoring the importance of local disease control and immunotherapeutic interventions (66).

## 3 The tumor immune microenvironment of melanoma

**Figure 2:**
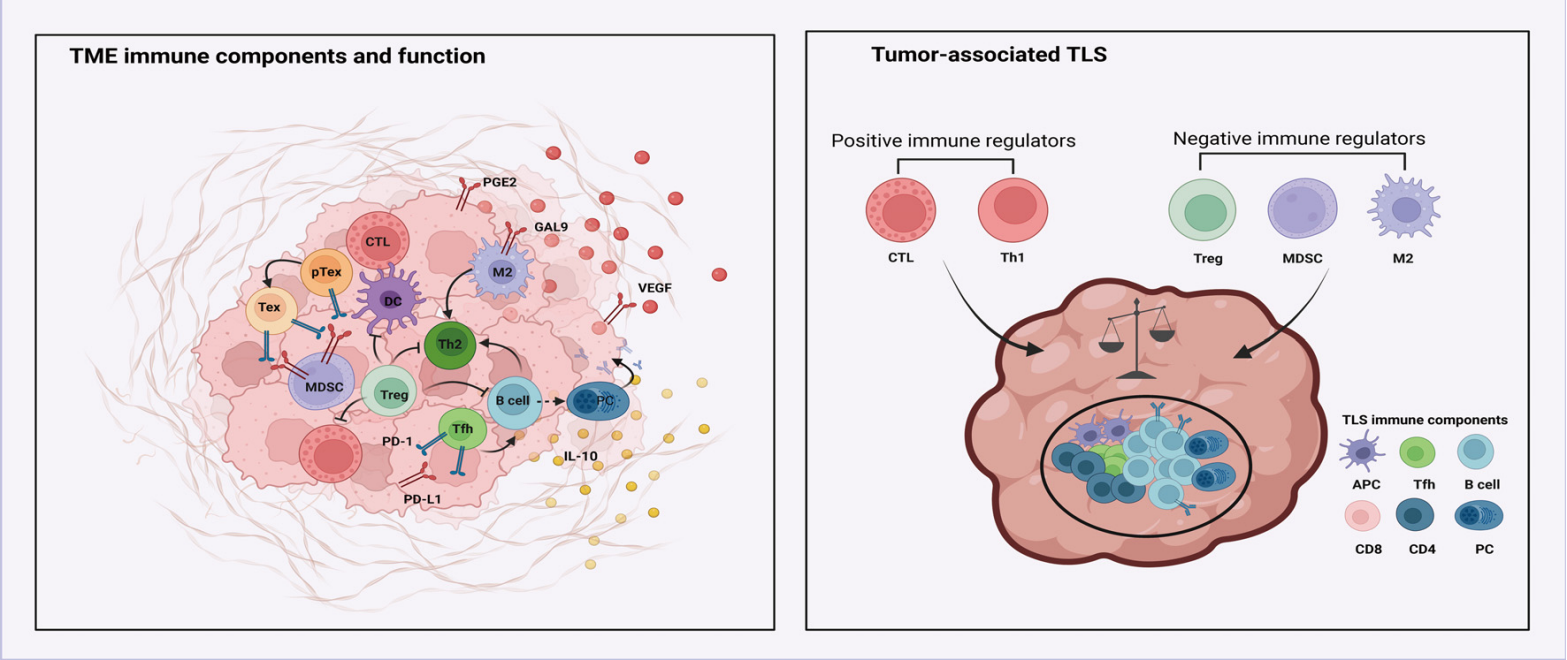
The immune microenvironment in melanoma.

The immune microenvironment of melanoma is a complex system that includes a variety of immune cells crucial for both tumor immune surveillance and suppression (**Figure 2**) (67, 68) (**Figure 2** **).** Among these cells, CD8+ T cells play a central role as effector cells responsible for directly eliminating melanoma cells (69). In contrast, CD4+ T cells exert regulatory control over immune responses (68), while natural killer (NK) cells function as vigilant sentinels, identifying and removing melanoma cells (70). Additionally, dendritic cells (DCs) act as pivotal coordinators, stimulating immune cell responses (71). Despite the collaborative actions of these immune effectors, melanoma employs immunosuppressive strategies mediated by tumor-associated macrophages (TAMs) that inhibit the function of other immune cells, demonstrating an immunosuppressive aspect within the tumor microenvironment (72). Moreover, regulatory T cells (Tregs), polymorphonuclear neutrophils (PMNs), and B cells play nuanced roles in the context of melanoma (25, 73). The interactions among these diverse immune cell populations are of utmost importance in the field of melanoma immunotherapy. Melanoma cells utilize sophisticated evasion mechanisms to evade immune surveillance by releasing immunosuppressive factors and diminishing antigen presentation capabilities. This evasion tactic hampers immune cell function, reducing their ability to recognize and eliminate tumor antigens, thereby facilitating tumor immune evasion.

Various cell types regulate and interact with the tumor microenvironment, including cytotoxic T lymphocytes (CTLs), dendritic cells (DCs), M2 type macrophages (M2), regulatory T cells (Tregs), exhausted T cells (Tex), myeloid-derived suppressor cells (MDSC), TH2 type helper T cells (TH2), T follicular helper cells (Tfh), and B cells. Each cell type is associated with different immune functions and interactions. For example, MDSCs interact with Tregs via the PD-L1 and PD-1 checkpoint pathway. Key molecules such as prostaglandin E2 (PGE2), vascular endothelial growth factor (VEGF), interleukin-10 (IL-10), and galectin-9 (GAL9) are indicated, showing their involvement in immune modulation. The tumor-associated tertiary lymphoid structures include positive immune regulators (CTLs and TH1 cells) and negative immune regulators (Tregs, MDSCs, and M2 macrophages).

ICIs have emerged as cornerstone therapeutics in the management of melanoma (74, 75). Among these, the most prominent are PD-1 and CTLA-4 inhibitors. PD-1 inhibitors function by impeding the interaction between the immune checkpoint molecule PD-1 and its ligand PD-L1, thereby thwarting the mechanism through which melanoma cells elude immune surveillance (76). Conversely, CTLA-4 inhibitors obstruct the CTLA-4 molecule on the surface of inhibitory tumor immune cells, bolstering the activity of immune effectors (77). The application of ICIs in melanoma therapy has yielded remarkable clinical efficacy. Comparative studies evince that, vis-à-vis traditional chemotherapy, ICIs not only prolong the survival duration of melanoma patients but also confer enduring therapeutic benefits whilst mitigating treatment-related adverse events. Nevertheless, ICIs prove efficacious in only a subset of patients, with some developing immune resistance. Consequently, ongoing research endeavors are earnestly pursuing novel ICIs or exploring combinatorial immunotherapeutic approaches to further augment treatment outcomes for melanoma patients.

## 4 Immunotherapy in MM

In recent years, substantial strides have been made in elucidating the immune mechanisms underpinning MM, catalyzing the development of innovative immunotherapeutic strategies. ICIs have emerged as promising agents capable of disrupting immune checkpoint interactions, thereby potently activating the host’s immune system and precipitating tumor cell destruction. In tandem with ICIs, modalities such as ACT, vascular endothelial growth factor (VEGF) inhibitors, and combination therapies have also demonstrated robust anti-tumor activities. The clinical trials investigating the efficacy of ICIs in MM are comprehensively outlined in **Table 1**. While The clinical trials investigating the efficacy of VEGF inhibitors in MM are comprehensively outlined in **Table 2**. These noteworthy advancements in immunotherapeutic approaches signify the advent of a new epoch in MM treatment.

**Table 1:** Summary of immunotherapy clinical trials targeting ICIs of patients with mucosal melanoma.

Number	Agent	Target	Status	Phazse	Primary Outcome Measures	Secondary Outcome Measures
NCT05420324	Pebolizumab/Albumin Paclitaxel	PD-1	RECRUITING	Phase II	Confirmed Objective Response Rate (ORR)	/
NCT04462965	Toripalimab/Temozolomide/Cisplatin	PD-1	RECRUITING	Phase II	RFS assessment per RECIST 1.1.	RFS assessment per RECIST1.1 in 1 year and 2 year
						DMFS assessed by investigator per RECIT1.1
						OS per death time
						Incidence and grade of AEs and SAEs related to study drugs
						per NCI-CTCAE version 5.0, AEs ≥ grade 3 related to the study drugs.
NCT04180995	Toripalimab/Axitinib	PD-1	UNKNOWN STATUS	Phase II	Pathological response (pCR+pPR) rate	RFS per RECIST1.1 as Assessed by investigator
						OS
						Incidence of AEs/SAEs
						Pathological complete response (pCR) rate
NCT04472806	Toripalimab	PD-1	UNKNOWN STATUS	Phase II	PFS	ORR
						OS
						Incidence of AEs/SAEs
NCT03178123	JS001	PD-1	ACTIVE, NOT RECRUITING	Phase II	RFS	DMFS
						RFS
						OS
						Number of participants with treatment-related adverse events
NCT03241186	Ipilimumab and Nivolumab	CTLA4/PD-1	ACTIVE, NOT RECRUITING	Phase II	RFS	Assess the Adverse Events
						OS
NCT03313206	Lenvatinib	PD-1	RECRUITING	Phase II	DFS	/
NCT05436990	Vactosertib/Pembrolizumab	PD-1	NOT YET RECRUITING	Phase II	ORR	
NCT06041724	Envafolimab/Endostatin	PD-L1	NOT YET RECRUITING	Phase II	PFS	ORR
						DCR
						DoR
						OS
						PD-L1 expression from the tissue and peripheral blood samples of the patients
						ctDNA expression from the tissue and peripheral blood samples of the patients
						AEs according to CTCAE v5.0.
NCT02978443	Ipilimumab	CTLA4	TERMINATED	Phase II	ORR With Mucosal Melanoma (MCM)	ORR with Acral Lentiginous Melanoma (ALM)
						PFS
						OS
NCT05111574	Nivolumab/Cabozantinib	PD-1	RECRUITING	Phase II	RFS	OS
						RFS
						PFS
						ORR
						Duration of response (Arm 3)
						Incidence of adverse events
NCT05089370	Oral Decitabine/​Cedazuridine/Nivolumab	PD-1	RECRUITING	Phase I/II	Determine the safety of DEC-C in combination with Nivolumab	Determine the response rate to DEC-C in combination with Nivolumab
					unresectable, locally advanced, or metastatic mucosal melanoma patients	in unresectable, locally advanced, or metastatic mucosal melanoma patients;
						Determine if the addition of DEC-C to Nivolumab increases PFS and OS in
						unresectable, locally advanced, or metastatic mucosal melanoma patients.
NCT04318717	Pembrolizumab	PD-1	RECRUITING	Phase II	Local tumor control rate	Number of treatment-related grade 3 or greater adverse events
						Number of treatment discontinuations due to treatment-related adverse events
						RFS
						DMFS
						OS

/, None.

**Table 2:** Summary of immunotherapy clinical trials targeting VEGF/VEGFR of patients with mucosal melanoma.

Number	Agent	Target	Status	Phase	Primary Outcome Measures	Secondary Outcome Measures
NCT05545969	Pembrolizumab/Lenvatinib	PD-1/VEGFR	NOT YET RECRUITING	Phase II	Change in immune cell expression of HIF1 and immune cell densities	RECIST response rate
					Pathological response rate	
NCT05384496	Axitinib/Nivolumab	VEGFR/PD-1	RECRUITING	Phase II	Best RECIST objective response	/
NCT03602547	CM082/JS001	VEGFR/PD-1	UNKNOWN STATUS	Phase II	ORR	Disease Control Rate
						Duration of Response
						Time to Response
						PFS
						OS
NCT06424626	AK104/AK112/Axitinib	VEGFR	NOT YET RECRUITING	Phase I	Number of participants with treatment-related	ORR by irRC and RECIST 1.1
					adverse events as assessed by CTCAE v4.0	Duration of Response (DOR) by irRC and RECIST 1.1
						Disease Control Rate (DCR) by irRC and RECIST 1.1
						Time to response (TTR) by irRC and RECIST 1.1
						PFS by irRC and RECIST 1.1
						OS by irRC and RECIST 1.1
NCT04622566	Lenvatinib/Pembrolizumab	VEGFR/PD-1	UNKNOWN STATUS	Phase II	Pathological complete response (pCR) rate	1 year RFS rate per RECIST1.1 as Assessed by investigator
						OS
						Incidence of AEs/SAEs
NCT02023710	Bevacizumab/Carboplatin/Paclitaxel	VEGF	UNKNOWN STATUS	Phase II	PFS	AE
						OS
NCT04091217	Atezolizumab/Bevacizumab	PD-L1/VEGF	COMPLETED	Phase II	ORR	PFS
						OS
						DOR
						DCR
						Percentage of Participants With Adverse Events

/, None.

### 4.1 ICIs

#### 4.1.1 PD-1 inhibitors

**Figure 3:**
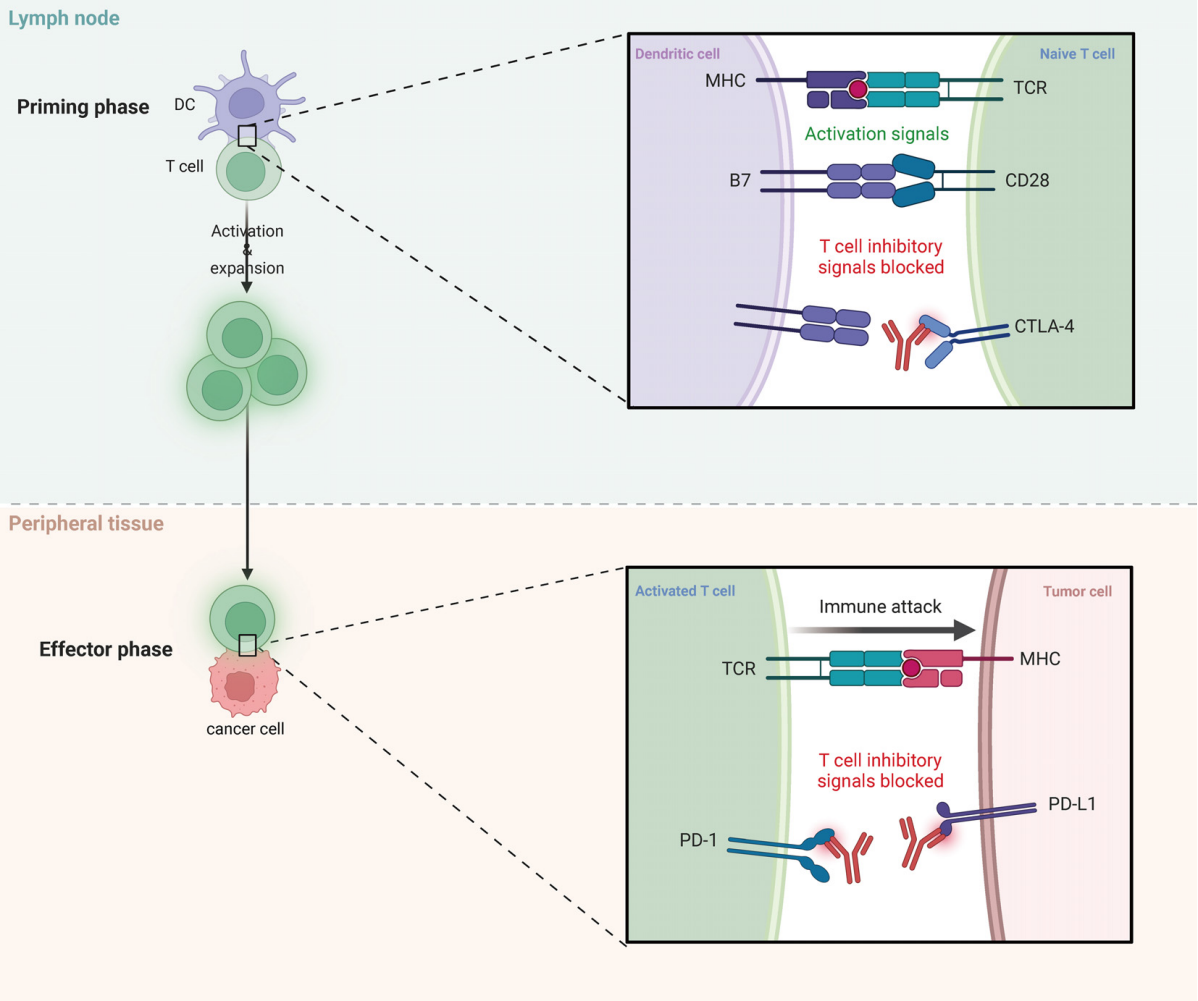
The mechanism of ICIs therapy.

PD-1 inhibitors comprise a pharmacological subclass of therapeutic agents designed to specifically target the immune checkpoint protein PD-1, thereby inhibiting the interaction between PD-1 and its ligands (**Figure 3**). This inhibition reinstates and amplifies the immune response of activated T cells against tumor cells (78, 79). Importantly, PD-1 inhibitors have exhibited considerable efficacy in patients with MM, as evidenced by a series of clinical trials validating their notable performance in MM therapy. These trials underscore the favorable and lasting responses achieved with PD-1 inhibitor monotherapy, providing survival benefits for individuals with advanced and metastatic melanoma. Promising outcomes include instances of complete remission or sustained periods free of disease progression. Thierauf et al. conducted an investigation into the effectiveness of anti-PD-1 therapy in patients with head and neck MM, evaluating the expression of Programmed Death-Ligand 1 (PD-L1) and PD-1 in tumor specimens. Despite the observed low levels of PD-L1 expression in these specimens, the study revealed that anti-PD-1 treatment did not elicit clinical responses in patients, highlighting the challenges and limitations associated with checkpoint inhibitors in the treatment of advanced MM (80). Similarly, Buchbinder et al. noted that MM displayed a relatively lower response rate (RR) to immune checkpoint blockade (ICB) compared to cutaneous melanoma. Targeted sequencing identified mutations in genes such as SF3B1, KIT, and NF1 in MM, but these genetic abnormalities did not significantly correlate with the response to ICB treatment (81). In a separate study, Ascierto et al. discerned that changes in lactate dehydrogenase (LDH) levels could serve as predictive markers of treatment response, with nivolumab demonstrating complete remission in certain patients. Despite the occurrence of manageable mild adverse events, the study emphasized the importance of identifying patients who are most likely to benefit from nivolumab monotherapy (82). Furthermore, Otsuka et al. revealed that patients experiencing immune-related adverse events (irAEs) during nivolumab therapy generally exhibited prolonged progression-free survival (PFS) and overall survival (OS), with the disease control rate (DCR) being significantly associated with the occurrence of irAEs. These findings suggest that irAEs could potentially serve as an effective biomarker for predicting treatment outcomes (83).

Indeed, while programmed cell death protein 1 (PD-1) inhibitors represent a promising therapeutic approach for the treatment of melanoma, not all patients experience optimal responses to these agents. Some individuals may demonstrate intrinsic resistance, while others may encounter adverse immune reactions. A case study by Sohail et al. focused on a female patient diagnosed with primary gastric mucosal melanoma (MMM) who exhibited an inadequate response to nivolumab, leading to a transition to palliative care. This case emphasized the infrequency and aggressive nature of primary gastric melanoma, highlighting the consideration of clinical trials for treatment-naive melanoma patients (84). Additionally, Zumelzu et al. reported a case of an 83-year-old patient who developed gingival mucosal erosions and blisters after discontinuing a 10-month pembrolizumab regimen for metastatic melanoma. Subsequent diagnosis revealed mild mucosal pemphigoid (MMP), which promptly achieved complete remission with minimal intervention (doxycycline). Noteworthy is the absence of melanoma recurrence 14 months post-pembrolizumab cessation, suggesting the possibility of unusual immune-related complications following PD-1 inhibitor therapy discontinuation. Furthermore, Nomura et al. conducted a study evaluating the efficacy and safety of nivolumab monotherapy for unresectable or metastatic melanoma, reporting an overall response rate (RR) of 23.5% (85). One patient achieved complete remission, three attained partial remission, and five experienced stable disease. The median progression-free survival (PFS) was 1.4 months, the median overall survival (OS) was 12.0 months, and the one-year OS rate was 50.0%. Notably, treatment-related grade 3 or 4 adverse events occurred in 15% of patients, managed effectively with corticosteroid therapy, highlighting the therapeutic potential of nivolumab despite associated adverse events (86).

#### 4.1.2 CTLA-4 inhibitors

CTLA-4 inhibitors exert their effects primarily through targeted inhibition of the CTLA-4 protein present on the cell surface, an essential modulator of T cell function (87, 88) (**Figure 3**). These inhibitors function by preventing the interaction between CTLA-4 and its ligands (B7-1 and B7-2), thereby eliminating the inhibitory signals on T cells, leading to enhanced anti-tumor immune responses (77, 89, 90). Initial clinical investigations have consistently shown notable therapeutic efficacy of CTLA-4 inhibitors in patients with MM. Saijo et al. performed a comprehensive analysis involving patients with advanced MM who had previously failed nivolumab therapy and subsequently received ipilimumab treatment. While complete or partial responses were not observed in any patients, disease stabilization was evident, with some individuals experiencing a reduction in tumor size. These findings highlight the potential of ipilimumab as an adjunct therapy that can provide additional benefits in terms of progression-free survival for patients with advanced MM refractory to nivolumab (91).

Immune checkpoints are critical molecules in the regulation of immune response cells, and tumor cells exploit these molecules to evade immune detection. The PD-1/PD-L1 and CTLA-4 pathways are significant immune checkpoint pathways with distinct mechanisms in cancer therapy. PD-1 primarily regulates immune responses by inhibiting T cell activity, whereas CTLA-4 predominantly regulates the initial activation of T cells in lymphoid tissue. CTLA-4 inhibits T cell activation and proliferation by competitively binding to B7 molecules, thereby reducing the binding of CD28 to B7. PD-1 inhibits T cell activation and function by binding to its ligand PD-L1, thus preventing excessive immune responses. Targeted therapies such as PD-1 inhibitors and CTLA-4 inhibitors can block these checkpoints, restore the anti-tumor activity of T cells, and enhance the immune system’s capacity to recognize and attack tumors.

Despite the demonstrated efficacy of CTLA-4 inhibitors in the treatment of melanoma, there are notable limitations and associated side effects to consider. Immune activation-related toxicities, such as immune-mediated side effects, can lead to severe adverse reactions in specific patient populations. Postow et al. conducted a retrospective analysis on patients with metastatic melanoma treated with ipilimumab, revealing immune-related responses that included complete response, partial response, stable disease, and progressive disease, albeit with an overall low response rate (RR) (92). Vecchio et al. assessed the efficacy of ipilimumab in metastatic melanoma patients, highlighting some response efficacy in addition to the ability to manage immune-related adverse events. Notably, treatment with ipilimumab resulted in improvements in progression-free survival (PFS) and overall survival (OS), though it was accompanied by a high incidence of treatment-related severe adverse events (93). Alexander et al. observed that melanoma patients treated with ipilimumab exhibited modest improvements in OS and PFS, with favorable one- and two-year survival rates. Factors such as melanoma subtype, lymphocyte count, and BRAF mutation status were identified as key influencers of treatment efficacy. However, despite comparable efficacy to clinical trials, the incidence of severe adverse events remained elevated (94). Additionally, Bello et al. reported that metastatic uveal melanoma patients undergoing ipilimumab treatment experienced symptoms resembling gastritis, initially mistaken for immune-related adverse events but indicative of an effective antitumor immune response against micro-metastases in the stomach. The study underscored the significance of a multidisciplinary approach in the management of patients receiving immunotherapy, particularly in cases involving atypical symptoms or rare etiologies (95).

### 4.2 ACT

Adoptive cell therapy (ACT) has emerged as a promising therapeutic approach in the field of melanoma treatment, attracting considerable attention (96). This innovative strategy involves the utilization of recombinant T cell receptors (TCR) or chimeric antigen receptors (CAR) to genetically modify a patient’s own T cells, equipping them with the capability to recognize and eliminate melanoma cells (97). Noteworthy is the personalized nature of this therapeutic modality, allowing for customization to individual patient profiles, thus enhancing treatment precision and effectiveness. Within the realm of melanoma, ACT has shown significant promise and considerable antitumor activity. Clinical trials utilizing engineered T cells targeting specific antigens, such as tumor-associated antigens (TAA) or tumor-specific antigens (TSA), have produced remarkable therapeutic results. Particularly in cases of metastatic or treatment-resistant melanoma, ACT has demonstrated encouraging long-term remissions and even complete clinical responses (98). Zhang et al. conducted an evaluation of the effectiveness and success rate of cytokine injection, cryosurgery, and adoptive cell transfer in managing patients with advanced oral MM. Their findings revealed objective clinical efficacy in all patients, including seven instances of sustained complete remission and three cases of partial remission, with five patients still alive. Notably, combined treatment led to an increase in the proportion of CD3+ lymphocytes and interferon-γ secretion, coupled with a decrease in interleukin-10 levels. Moreover, improved cytokine-induced killer cell assays showcased a decline in CD4+CD25+ Tregs alongside an upregulation of NK cells. Additionally, the proliferation rate of modified cytokine-induced killer cells cultured *in vitro* exhibited enhancement post-treatment (99).

### 4.3 Anti-angiogenic therapy

Anti-angiogenic therapy plays a crucial role in pharmacological intervention to impede the growth and metastasis of tumors by inhibiting angiogenesis. Within the context of melanoma, this therapeutic approach has shown promise and effectiveness. Research highlights the enhanced angiogenic capacity of MM compared to other types of melanoma, providing a basis for exploiting anti-angiogenic therapy (100). This therapeutic modality targets key signaling pathways that are essential for tumor angiogenesis, specifically the vascular endothelial growth factor-vascular endothelial growth factor receptor (VEGF-VEGFR) pathway, in order to disrupt the formation of tumor blood vessels. Consequently, anti-angiogenic therapy reduces tumor growth and blood supply, thus inhibiting tumor proliferation and spread (101, 102). Clinical trials have investigated the application of anti-angiogenic therapy in MM, assessing the efficacy of anti-angiogenic drugs as standalone interventions and in combination with other modalities such as immunotherapy (103). While ongoing research is still in the early stages, evidence suggests the effectiveness of anti-angiogenic therapy in MM. However, this therapeutic approach is accompanied by challenges and side effects, including hypertension, bleeding risk, and potential development of resistance. Moreover, the significant heterogeneity of MM may result in diverse responses to anti-angiogenic therapy among different cases. Therefore, further comprehensive research and clinical trials are necessary to fully understand the potential of anti-angiogenic therapy in MM and determine the optimal treatment strategy.

### 4.4 Combination therapies

Emerging evidence indicates that the use of PD-1 monotherapy in patients with MM frequently confronts a substantial obstacle characterized by primary resistance. In order to tackle this dilemma, emphasis has shifted towards combination therapies that employ PD-1 inhibitors as a foundational element. By integrating supplementary agents that target distinct pathways, such as other checkpoint inhibitors and targeted therapies, there exists the potential to surmount primary resistance and enhance treatment outcomes.

#### 4.4.1 Anti−PD−1 combined with other ICIs

Shustef et al. observed a regression of pigmented lesions in the gastric mucosa following ICB therapy, indicating the potential of ICIs such as ipilimumab and pembrolizumab in the treatment of advanced melanoma. It is worth noting that primary gastric melanoma is a rare condition with generally poor prognosis. Nevertheless, these treatments have been shown to enhance anti-tumor activity (104). In a study conducted by Plana et al. (105), the response of MM patients to anti-CTLA4 and anti-PD1 immunotherapy was evaluated. The findings revealed that the objective response rate (RR) and progression-free survival (PFS) were comparable to those observed in cutaneous melanoma patients. Notably, pembrolizumab demonstrated a more favorable risk-benefit ratio. Umeda et al. (106) treated multiple patients with advanced MM using anti-PD-1 monotherapy or a combination of anti-PD-1 and anti-CTLA-4 therapy. The addition of radiotherapy did not significantly impact the objective RR, PFS, or overall survival (OS). It was concluded that while radiotherapy can improve local tumor control and alleviate symptoms, it does not extend the survival of patients with advanced MM. A case study by Sezen et al. (107) reported a patient with metastatic vaginal MM who achieved complete remission through dual checkpoint inhibitor immunotherapy combined with high-dose and low-dose radiotherapy. The treatment regimen included ipilimumab and nivolumab. The pathological features showed characteristics of mucosal melanoma (MM), and staging and genetic alterations were taken into consideration. Lymphocyte-activation gene 3 (LAG-3) is an immune checkpoint protein that inhibits T cell activity and is upregulated in various tumor types, including melanoma. Preclinical models have demonstrated synergistic effects when combining anti-LAG3 and anti-PD-1 treatments (108). In the recently presented RELATIVITY-047 phase II/III study (109), the combination of the LAG-3 inhibitor relatlimab with nivolumab exhibited promising efficacy, particularly in patients with cutaneous melanoma. Other potential combinations that show promise include targeting T cell immunoglobulin and mucin-domain-containing-3 (TIM-3) and glucocorticoid-induced tumor necrosis factor receptor (GITR).

#### 4.4.2 Anti−PD−1/PD−L1 combined with targeted therapy agents

A phase Ib study examining the combination of toripalimab and axitinib in patients with metastatic melanoma demonstrated notable responses (110). Among a cohort of 29 treatment-naïve individuals, the overall response rate (ORR) was 48.3%, and the disease control rate (DCR) was 86.2%. The median duration of response (DOR) was 13.7 months, with a median progression-free survival (PFS) of 7.5 months and overall survival (OS) of 20.7 months. Of interest, there was no significant association between programmed death-ligand 1 (PD-L1) expression or tumor mutational burden (TMB) and treatment response. However, a strong correlation was observed between gene expression programming (GEP) scores of certain immune-related and angiogenesis-related genes and treatment response. Building on these findings, a randomized three-arm phase II trial has been initiated to compare the toripalimab/axitinib combination with toripalimab or axitinib monotherapy. In another phase II study, the combination of vorolanib with toripalimab yielded an ORR of 22.2%, a DCR of 55.5%, and a median PFS of 5.7 months (111). Additionally, encouraging results were observed with the combination of lenvatinib and pembrolizumab in melanoma, renal cell carcinoma, and endometrial cancer. These findings have prompted the initiation of an ongoing international multicenter phase III trial for the treatment of metastatic melanoma (112). Moreover, the combination of atezolizumab, an anti-PD-L1 inhibitor, with the vascular endothelial growth factor receptor (VEGFR) monoclonal antibody bevacizumab demonstrated a favorable ORR of 36.4% and a tolerable safety profile in patients with advanced melanoma (113). Heppt et al. presented a case report of a male patient with nivolumab-refractory unresectable melanoma who received intralesional injections of interleukin-2 (IL-2) (114). After six months, a notable reduction in tumor size was observed. This study underscores the potential efficacy of intralesional IL-2 therapy and highlights the underutilized opportunities for intratumoral treatment modalities.

#### 4.4.3 Anti−PD−1/PD−L1 combined with other candidates

Adileh et al. analyzed the effects of ICI therapy in patients undergoing surgical resection of anorectal melanoma. The results indicated that ICI therapy did not significantly enhance patient survival, and no clinical or pathological features were identified that correlated with ICI treatment response or survival outcomes. The study suggests that ICI therapy alone may not improve survival rates in anorectal melanoma patients, highlighting the need for further research into combination therapies (115). KIMURA et al. found that for female patients treated with nivolumab and undergoing debulking surgery, the combination of debulking surgery with ICI could offer potential benefits. Specifically, for patients with large tumors at the start of treatment or those whose tumors grew after ICI therapy, this combined approach could enhance response and prolong the duration of the response. Additionally, debulking surgery might enhance the efficacy of ICI by reducing tumor burden and inducing abscopal effects (116). Beyond the aforementioned combinations, other agents are being investigated for their potential to augment the efficacy of anti-PD-1 antibodies in melanoma. These include epigenetic regulators such as EZH2, DNA methyltransferase (DNMT), and histone deacetylase (HDAC), as well as oncolytic virotherapies and toll-like receptor 9 (TLR9) agonists. Additionally, engineered cytokines capable of modulating the tumor microenvironment (TME) have shown promising antitumor activity both as monotherapies and in combination with pembrolizumab. These innovative approaches hold significant potential for improving treatment outcomes in melanoma patients (117). Tang et al. found that the combination of axitinib and anti-PD-1 antibody therapy was effective for treating MM. The efficacy in patients with liver metastases treated with transcatheter arterial chemoembolization (TACE) varied and was associated with LDH levels and Eastern Cooperative Oncology Group (ECOG) status. The study results suggest that combination therapy is suitable for advanced MM, especially as a first-line treatment, and that TACE might improve treatment outcomes (118).

## 5 Discussion and prospects

**Figure 4:**
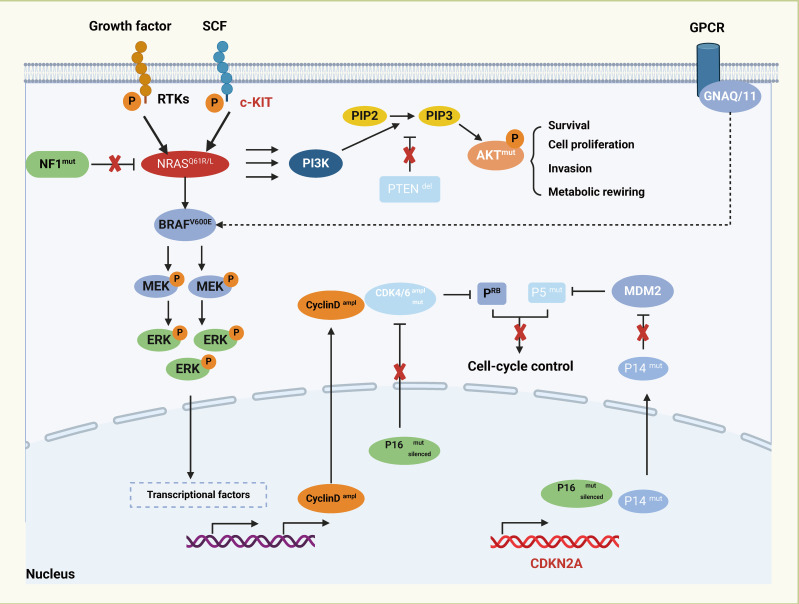
The pros and cons of neoadjuvant therapy.

Melanoma represents a rare and highly aggressive tumor, presenting significant challenges in both therapeutic approaches and research endeavors. Nevertheless, the continuous progress in scientific and technological realms, accompanied by a deepened comprehension of this condition, are facilitating advancements towards enhanced MM management and treatment strategies. The combination of surgical interventions alongside systemic therapies has demonstrated efficacy in facilitating the control of disease progression and metastatic dissemination. Neoadjuvant therapy, which entails the administration of therapeutic agents prior to the primary tumor-directed treatment, offers various benefits. Principally, this approach can lead to tumor size reduction, enabling less extensive surgical resections and the potential preservation of vital anatomical structures. Moreover, this preoperative maneuver permits early evaluation of tumor responsiveness to the therapeutic regimen, thereby informing subsequent treatment modalities. Additionally, neoadjuvant therapy holds the potential to eradicate micrometastatic foci, thereby enhancing overall survival outcomes. Nonetheless, noteworthy drawbacks exist. The deferment of definitive surgery could prove deleterious if the tumor fails to exhibit favorable responses to the prescribed therapy, consequently allowing for disease progression. Furthermore, adverse effects stemming from the therapeutic agents may compromise the patient’s general well-being, consequently complicating subsequent surgical procedures. Moreover, the optimal timing and combination of neoadjuvant therapies remain subjects of ongoing investigation across various cancer types, necessitating a delicate balance between prospective benefits and associated risks. These considerations underscore the imperative for individualized treatment strategies and the necessity for continued research efforts. The benefits and drawbacks of neoadjuvant therapy are illustrated in **Figure 4**.

The potential impacts of neoadjuvant therapy include the following: It may elicit a more extensive and potent immune response due to a broader array of tumor antigens. Increased intratumoral immune infiltration is associated with a pathological response and extended event-free survival (EFS), as immune cells migrate to potential micrometastases elsewhere in the body. Reducing tumor size may facilitate easier surgical removal. Additionally, assessing the pathological response post-treatment provides a rapid indication of the treatment’s effectiveness. However, surgery might be complicated by immune checkpoint blockade (ICB) side effects or delayed due to side effects or their management. Furthermore, disease progression can complicate surgery or render the tumor inoperable.

In recent years, significant progress has been made in the field of immunotherapy for the treatment of melanoma (119, 120). Immune checkpoint inhibitors (ICIs), such as PD-1 inhibitors and CTLA-4 inhibitors, have emerged as promising therapeutic agents by enhancing the host’s immune response against MM (121, 122). Clinical studies have demonstrated positive responses and favorable disease control with the administration of PD-1 and CTLA-4 inhibitors, leading to prolonged survival in MM patients. Additionally, cellular immunotherapy, involving the modification of T cells to specifically target and eliminate melanoma cells, has shown potential as an immunotherapeutic strategy. Although these approaches are currently at experimental stages, initial results suggest encouraging therapeutic possibilities. Furthermore, the combination of immunotherapy with conventional drug therapies, such as targeted therapy, has been investigated. The co-administration of immunotherapeutic drugs like ICIs with BRAF inhibitors or MEK inhibitors has shown substantial improvement in treatment response rates and overall survival of MM patients (123). In conclusion, significant advancements in the field of immunotherapy have been witnessed in the management of Mucosal melanoma (MM). Though the current stage of research represents the early phases, the accruing results provide a beacon of hope for patients, thus emphasizing the imperative nature of further exploration and refinement of treatment modalities for enhanced efficacy. Notwithstanding the strides made in MM treatment and investigative endeavors, a spectrum of challenges and complexities persists. These obstacles encompass the issue of resistance to immunotherapeutic interventions and the pressing necessity to ameliorate the prognosis and overall survival rates among individuals diagnosed with MM. While immunotherapy exhibits a degree of efficacy, its universal applicability is not absolute, with instances of patients developing resistance or tolerance to Immune Checkpoint Inhibitors (ICIs) and other immune-based pharmaceuticals. Hence, the pivotal direction for research lies in the realms of predicting patient responsiveness to immunotherapy, coupled with a profound comprehension of the underlying biochemical pathways involved. Furthermore, the combination of immunotherapeutic agents, cellular therapies, and radiotherapy presents a promising avenue for future investigation to bolster patient response to treatment. Additionally, the enhancement of survival outcomes and prognostic indicators for MM patients stands as a formidable challenge. Despite notable successes in immunotherapeutic advancements, a sizeable cohort of MM patients grapple with the looming risks of disease recurrence and metastasis, hence resulting in unfavorable prognoses. Thus, delving into the intricate molecular frameworks and the disease progression dynamics of MM assumes critical importance. This knowledge reservoir will be instrumental in sculpting tailor-made and precise therapeutic approaches, thereby fostering improved clinical outcomes for patients.

In conclusion, the treatment and research of MM still face significant challenges. However, with the advancement of science and technology, as well as deeper research, the understanding of MM and the available treatment methods will continue to improve. Our collective efforts aim to achieve more accurate early diagnosis, develop more effective treatment strategies, and improve prognoses.

## Author contributions

ZS: Writing – original draft. FL: Writing – original draft, Writing – review & editing.

## Glossary

MM: Mucosal melanoma
ICIs: Immune checkpoint inhibitors
MMM: mucosal malignant melanoma
SNMM: sinonasal MM
NK: natural killer
DCs: Dendritic cells
TAMs: tumor-associated macrophages
Tregs: regulatory T cells
PMNs: Polymorphonuclear Neutrophils
PD-L1: Programmed Death-Ligand 1
ICB: immune checkpoint blockade
LDH: lactate dehydrogenase
irAEs: immune-related adverse events
MMP: mild mucosal pemphigoid
RR: response rate
PFS: progression-free survival
ACT: Adoptive cell therapy
TCR: T cell receptors
CAR: chimeric antigen receptors
TAA: tumor-associated antigens
TSA: tumor-specific antigens
CTLA-4: Cytotoxic T lymphocyte-associated protein 4
LAG-3: Lymphocyte-activation gene 3
TIM-3: mucin-domain-containing-3
GITR: glucocorticoid-induced tumor necrosis factor receptor
ORR: overall response rate
DCR: disease control rate
DOR: duration of response
OS: overall survival
TMB: tumor mutational burden
GEP: gene expression programming
IL-2: interleukin-2
DNMT: DNA methyltransferase
HDAC: histone deacetylase
TLR9: toll-like receptor 9
TME: tumor microenvironment
TACE: transcatheter arterial chemoembolization
ECOG: Eastern Cooperative Oncology Group
PD-1: Programmed Death-1
BRAF: B-Raf Proto-Oncogene, Serine/Threonine Kinase
KIT: KIT Proto-Oncogene, Receptor Tyrosine Kinase
NRAS: Neuroblastoma RAS Viral Oncogene Homolog
NF1: neurofibromatosis type-1
SF3B1: Splicing Factor 3b, Subunit 1
RAF: Rapidly Accelerated Fibrosarcoma
MEK: Mitogen-Activated Protein Kinase/Extracellular Signal-Regulated Kinase Kinase
ERK: Extracellular Signal-Regulated Kinase
MAPK: Mitogen-Activated Protein Kinase
TP53: Tumor Protein p53
CDKN2A: Cyclin-Dependent Kinase Inhibitor 2A
PTEN: Phosphatase and Tensin Homolog
VEGF: vascular endothelial growth factor
VEGFR: vascular endothelial growth factor receptor
